# Potential Sexual Transmission of Zika Virus

**DOI:** 10.3201/eid2102.141363

**Published:** 2015-02

**Authors:** Didier Musso, Claudine Roche, Emilie Robin, Tuxuan Nhan, Anita Teissier, Van-Mai Cao-Lormeau

**Affiliations:** Institut Louis Malardé, Papeete, Tahiti, French Polynesia

## Abstract

In December 2013, during a Zika virus (ZIKV) outbreak in French Polynesia, a patient in Tahiti sought treatment for hematospermia, and ZIKV was isolated from his semen. ZIKV transmission by sexual intercourse has been previously suspected. This observation supports the possibility that ZIKV could be transmitted sexually.

Zika virus (ZIKV) is a mosquitoborne arbovirus in the family *Flaviviridae*, genus *Flavivirus*. It was first isolated in 1947 from a rhesus monkey in the Zika forest of Uganda (*1*). Sporadic human cases were reported from the 1960s in Asia and Africa. The first reported large outbreak occurred in 2007 on Yap Island, Federated States of Micronesia (*2*). The largest known ZIKV outbreak reported started in October 2013 in French Polynesia, South Pacific (*3*), a territory of France comprising 67 inhabited islands; an estimated 28,000 persons (11% of the population) sought medical care for the illness (*4*). The most common symptoms of Zika fever are rash, fever, arthralgia, and conjunctivitis. Most of the patients had mild disease, but severe neurologic complications have been described in other patients in French Polynesia (*5*).

## The Study

In early December 2013, during the ZIKV outbreak, a 44-year-old man in Tahiti had symptoms of ZIKV infection: asthenia, low grade fever (temperature from 37.5°C to 38°C) and arthralgia. Symptoms lasted 3 days. Eight weeks later, he described a second episode of symptoms compatible with ZIKV infection: temperature from 37.5°C to 38°C, headache on days 1–3, and wrist arthralgia on days 5–7. The patient did not seek treatment, thus biological samples were not collected during the first 2 periods of illness. The patient fully recovered from the second episode, but 2 weeks later he noted signs of hematospermia and sought treatment. Because the patient had experienced symptoms of ZIKV infection some weeks before, he was referred to our laboratory in the Institut Louis Malardé, Papeete, Tahiti for ZIKV infection diagnostic testing. The medical questionnaire revealed no signs of urinary tract infection, prostatitis, urethritis, or cystitis, and the patient stated that he did not had any recent physical contact with persons who had acute ZIKV infection. We collected blood and semen samples. Direct and macroscopic examinations of the semen confirmed hematospermia. We extracted RNA using the NucliSENS easyMAG system (bioMérieux, Marcy l’Etoile, France) from 200 μL of blood and from 500 μL of semen and urine; both were eluted by 50 μL of elution buffer. We used 5 μL of RNA extracted for amplification. We tested blood and semen RNA extracts using real-time reverse transcription PCR (rRT-PCR) as described using 2 primers/probe amplification sets specific for ZIKV (*3*). The rRT-PCR results were positive for ZIKV in semen and negative in blood, and confirmed by sequencing of the genomic position 858–1138 encompassing the prM/E protein coding regions of ZIKV. The generated sequence (GenBank accession no. KM014700) was identical to those previously reported at the beginning of the ZIKV outbreak (*3*). Three days later, we collected a urine sample, then a second set of blood and semen samples. Semen and urine from this second collection were not found to contain traces of blood by both direct and macroscopic examinations. rRT-PCR detected ZIKV RNA in the semen and urine, but not in the blood sample.

We quantified ZIKV RNA loads using an RNA synthetic transcript standard that covers the region targeted by the 2 primers/probe sets. RNA loads were: 2.9 × 10^7^ copies/mL and 1.1 × 10^7^ copies/mL in the first and second semen samples, respectively, and 3.8 × 10^3^ copies/mL in the urine sample.

We cultured semen and urine as described for dengue virus cultured from urine (*6*). Briefly, 200 μL of each sample diluted in 200 μL of 1% fetal calf serum (FCS) minimum essential medium (MEM) were inoculated onto Vero cells and incubated for 1 h at 37°C; inoculum was then removed and replaced by 1 mL of culture medium. We also inoculated a negative control (200 μL of 1% FCS-MEM) and a positive control (5 μL of a ZIKV-positive serum diluted in 200 μL of 1% FCS-MEM). The cells were then incubated at 37°C in 5% CO_2_ for 6 days. The presence of ZIKV in the culture fluids was detected by rRT-PCR as described.

Replicative ZIKV particles were found in the 2 semen samples but none were detected in the urine sample. This finding does not exclude the possibility that ZIKV particles were present in urine. Positive samples were not titered.

## Conclusions

The ZIKV natural transmission cycle involves mosquitoes, especially *Aedes* spp. (*7*), but perinatal transmission (*8*) and potential risk for transfusion-transmitted ZIKV infections has also been demonstrated (*9*). Moreover, ZIKV transmission by sexual intercourse has been suggested by Foy et al. (*10*), who described a patient who was infected with ZIKV in southeastern Senegal in 2008. After returning to his home in Colorado, United States, he experienced common symptoms of ZIKV infection and symptoms of prostatitis. Four days later, he observed signs of hematospermia, and on the same day, his wife had symptoms of ZIKV infection. Because the wife of the patient had not traveled out of the United States during the previous year and had sexual intercourse with him 1 day after he returned home, transmission by semen was suggested. ZIKV infection of the patient and his wife was confirmed by serologic testing, but the presence of ZIKV in the semen of the patient was not investigated.

Infectious organisms, especially sexually transmitted microorganisms including viruses (human papillomavirus or herpes simplex virus), are known to be etiologic agents of hematospermia (*11*). To our knowledge, before the report of Foy et al. (*10*) and this study, arbovirus infections in humans had not been reported to be associated with hematospermia, and no arboviruses had been isolated from human semen.

We detected a high ZIKV RNA load and replicative ZIKV in semen samples, but ZIKV remained undetectable by rRT-PCR in the blood sample collected at the same time. These results suggest that viral replication may have occurred in the genital tract, but we do not know when this replication started and how long it lasted. The fact that the patient had no common symptoms of ZIKV acute infection concomitantly to hematospermia suggests that the viremic phase occurred upstream, probably during the first or second episode of mild fever, headache, and arthralgia.

The detection of ZIKV in both urine and semen is consistent with the results obtained in a study of effects of Japanese encephalitis virus, another flavivirus, on boars. The virus was isolated from urine and semen of experimentally infected animals, and viremia developed in female boars that artificially inseminated with the infectious semen (*12*).

Flaviviruses have also been detected in urine of persons infected with West Nile virus (WNV). WNV RNA was detected in urine for a longer time and with higher RNA load than in plasma (*13*). WNV antigens were detected in renal tubular epithelial cells, vascular endothelial cells, and macrophages of kidneys from infected hamsters (*14*), suggesting that persistent shedding of WNV in urine was caused by viral replication in renal tissue. Dengue virus (DENV) RNA and DENV nonstructural protein 1 antigen were also detected in urine samples for a longer time than in blood, but infectious DENV has not been isolated in culture. Hirayama et al. concluded that the detection of DENV by rRT-PCR was useful to confirm DENV infections after the viremic phase (*6*). Also, yellow fever virus RNA was isolated from the urine of vaccinated persons (*15*), and Saint Louis encephalitis viral antigens, but not infective virus, have been detected in urine samples from infected patients (*10*).

Our findings support the hypothesis that ZIKV can be transmitted by sexual intercourse. Furthermore, the observation that ZIKV RNA was detectable in urine after viremia clearance in blood suggests that, as found for DENV and WNV infections, urine samples can yield evidence ofZIKV for late diagnosis, but more investigation is needed.
