# Correction: Vol. 21, No. 2

**DOI:** 10.3201/eid2103.C12103

**Published:** 2015-03

**Keywords:** errata, erratum, correction

The abbreviation mL was inadvertently used in the place of μL in paragraphs 2 and 4 in the article Potential Sexual Transmission of Zika Virus (D. Musso et al.) The article has been corrected online (http://wwwnc.cdc.gov/eid/article/21/2/14-1363_article.htm).

